# The Role of Phlebovirus Glycoproteins in Viral Entry, Assembly and Release

**DOI:** 10.3390/v8070202

**Published:** 2016-07-21

**Authors:** Martin Spiegel, Teresa Plegge, Stefan Pöhlmann

**Affiliations:** 1 Infection Biology Unit, Deutsches Primatenzentrum, Kellnerweg 4, Göttingen 37077, Germany; TPlegge@dpz.eu; 2 Institute for Microbiology and Virology, Medizinische Hochschule Brandenburg Theodor Fontane, Grossenhainer Str. 57, Senftenberg 01968, Germany

**Keywords:** Bunyaviridae, phlebovirus, glycoproteins, virus attachment, entry, membrane fusion, signal peptidase, assembly

## Abstract

Bunyaviruses are enveloped viruses with a tripartite RNA genome that can pose a serious threat to animal and human health. Members of the *Phlebovirus* genus of the family *Bunyaviridae* are transmitted by mosquitos and ticks to humans and include highly pathogenic agents like Rift Valley fever virus (RVFV) and severe fever with thrombocytopenia syndrome virus (SFTSV) as well as viruses that do not cause disease in humans, like *Uukuniemi* virus (UUKV). Phleboviruses and other bunyaviruses use their envelope proteins, Gn and Gc, for entry into target cells and for assembly of progeny particles in infected cells. Thus, binding of Gn and Gc to cell surface factors promotes viral attachment and uptake into cells and exposure to endosomal low pH induces Gc-driven fusion of the viral and the vesicle membranes. Moreover, Gn and Gc facilitate virion incorporation of the viral genome via their intracellular domains and Gn and Gc interactions allow the formation of a highly ordered glycoprotein lattice on the virion surface. Studies conducted in the last decade provided important insights into the configuration of phlebovirus Gn and Gc proteins in the viral membrane, the cellular factors used by phleboviruses for entry and the mechanisms employed by phlebovirus Gc proteins for membrane fusion. Here, we will review our knowledge on the glycoprotein biogenesis and the role of Gn and Gc proteins in the phlebovirus replication cycle.

## 1. Introduction

The family *Bunyaviridae* comprises over 350 viruses, which infect diverse animals, insects, and plants. Five *Bunyavirus* genera have been identified: *Orthobunyavirus*, *Hantavirus*, *Nairovirus*, *Phlebovirus* and *Tospovirus* based on serologic, morphologic and biochemical criteria [1]. Viruses within the *Orthobunyavirus*, *Nairovirus* and *Phlebovirus* genera are transmitted to animal hosts by arthropod vectors, such as ticks, mosquitoes, midges, and flies during blood meals [2]. Tospoviruses also employ arthropods and thrips for spread but infect plants [3]. In contrast, hantaviruses infect rodents, bats, shrews, and moles [4,5,6,7,8,9,10] and are transmitted to humans upon exposure to aerosolized rodent excreta [2,11]. Several bunyaviruses cause severe disease, including hemorrhagic fevers in humans, and are teratogenic in animals. In addition, many bunyaviruses are “emerging”, since disease incidence and geographical distribution are increasing. Thus, bunyaviruses can pose a significant threat to human health and understanding how these viruses replicate, spread, and cause disease is required to identify targets for intervention.

Bunyaviruses are enveloped viruses which harbor a tripartite, single stranded RNA genome with negative polarity. The l-segment of the genome encodes for the viral polymerase (L), the M-segment for the viral glycoproteins, Gn and Gc, and the S-segment for the nucleocapsid (N) protein [12]. In addition, non-structural proteins can be encoded by the S- and M-segment, employing either an ambisense coding strategy, overlapping open reading frames or an open reading frame (ORF) encoding a polyprotein. The glycoproteins mediate the first step in the bunyavirus replication cycle—viral entry into host cells— and are the only targets for neutralizing antibodies. Gn and Gc are synthesized as a precursor protein, Gn/Gc, in the secretory pathway of infected cells. Gn and Gc are separated by proteolytic cleavage but may remain non-covalently associated [13,14]. The cleavage step is executed by a cellular enzyme, signal peptidase [15,16,17], during import of the Gn/Gc precursor into the endoplasmic reticulum (ER). In the ER, Gn and Gc are decorated with *N*-linked glycans [18,19] of the high-mannose type, which can be processed into hybrid and complex forms upon import of Gn and Gc into the Golgi apparatus [18,19,20,21]. The Golgi apparatus is the site of bunyavirus budding [22,23,24,25,26] and this process is facilitated by Gn and Gc, which play a key role in particle morphogenesis and genome incorporation [27,28,29,30,31]. Finally, infectious particles decorated with Gn and Gc are released from the infected cell by exocytosis.

**Figure 1:**
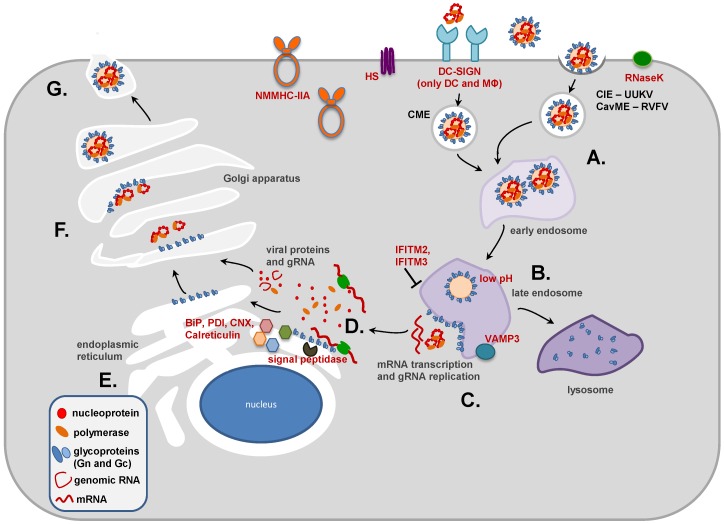
Replication cycle of phleboviruses. (**A**) Cellular attachment of phleboviruses is driven by glycoprotein interactions with host cell factors such as dendritic cell-specific intercellular adhesion molecule-3-grabbing non-integrin (DC-SIGN), heparan sulfate (HS), or non-muscle myosin heavy chain IIA (NMMHC-IIA). The binding to DC-SIGN and so far unknown entry factors induces uptake via caveolin-1-mediated endocytosis (CavME) (as for Rift Valley fever virus, RVFV) or incompletely defined clathrin-independent endocytic (CIE) mechanisms (as for Uukuniemi virus, UUKV). Ribonuclease kappa (RNaseK) promotes the internalization of virions by a yet unknown mechanism; (**B**) In late endosomes, the low pH induces the membrane fusion activity of the Gc protein. Expression of vesicle-associated membrane protein 3 (VAMP3) promotes UUKV penetration, while interferon-induced transmembrane protein (IFITM) 2 and IFITM3 inhibit the fusion of RVFV in late endosomes; (**C**) The fusion of viral and endosomal membranes allows release of the viral ribonucleoprotein complexes into the cytoplasm, the site of viral transcription and replication; (**D**) The viral glycoproteins Gn and Gc are translated at the rough endoplasmic reticulum (ER) as a precursor protein, Gn/Gc, which is cleaved by signal peptidase. The viral nucleoprotein and the viral polymerase are synthesized in the cytoplasm where they form together with newly produced genomic RNA (gRNA) ribonucleoprotein (RNP) complexes; (**E**) Binding immunoglobulin protein (BiP) and calnexin, two ER chaperones, are required for appropriate folding of Gn and Gc. Similarly, protein-disulfide-isomerase catalyzes Gn and Gc folding by promoting the formation of disulfide bonds, while calreticulin prevents misfolded Gn and Gc from being exported from the ER to the Golgi; (**F**) Correctly folded Gn/Gc heterodimers are transported into the Golgi apparatus where they associate with RNPs via the cytoplasmic tails of Gn during the budding process; (**G**) After budding of new virus particles into the Golgi is complete, virus-containing vesicles are transported to the plasma membrane where the virions are released by exocytosis. DC: dendritic cell; MФ: macrophage; CME: clathrin-mediated endocytosis; PDI: protein disulfide isomerase; CNX: calnexin.

Despite their important role in bunyavirus entry and release, biogenesis and biological activities of bunyavirus Gn and Gc proteins are incompletely understood. In the present manuscript, we will review our knowledge on phlebovirus glycoproteins. The genus *Phlebovirus* (*Phlebotominae*, sandflies) currently contains 10 species, with *Rift Valley fever virus* (*RVFV*) being the type species, and the viruses grouped into the sandfly fever virus (SFV) and the Uukuniemi virus (UUKV) groups, depending on their vector species. Several phleboviruses are important human pathogens: RVFV causes severe diseases in ruminants and humans in Africa and the Middle East [32] while severe fever with thrombocytopenia syndrome virus (SFTSV) was discovered as a novel agent responsible for cases of severe fever in Asia, which may take a fatal course particularly in elderly patients [33,34]. In contrast, UUKV is not pathogenic in humans. For further information on phlebovirus biology and disease in general, the reader is referred to recent reviews [35,36]. Here, we will discuss how the glycoproteins of phleboviruses are generated and how they promote virus entry and release. For this, we will describe the role of the glycoproteins at different stages of the viral replication cycle, starting with their configuration in the envelope of infectious particles, followed by their function during viral entry, their biogenesis in infected cells and finally their roles during assembly and release of progeny phlebovirus particles (see Figure 1).

## 2. Role of Gn and Gc in Phlebovirus Entry

### 2.1. Configuration of Gn and Gc Proteins in the Viral Envelope

Initial studies provided evidence that bunyavirus particles are pleomorphic [37]. It was therefore surprising that electron cryotomography revealed that both UUKV [38] and RVFV [39,40] particles display a spherical, highly ordered structure. The order is imposed by the configuration of Gn and Gc proteins in the viral envelope, which form an icosahedral lattice with a triangulation number of 12 [38,39,40]. The lattice is composed of 110 hexameric and 12 pentameric capsomers, and for RVFV it was proposed that the capsomers accommodate in total 720 Gn/Gc heterodimers [41,42], with Gn forming the capsomer spikes while Gc lies partially underneath, closer to the lipid membrane. The shape of the capsomers depends on the pH of the surrounding medium [38], since protonation triggers major conformational changes in Gc, which are associated with membrane fusion, as discussed below. Since the RVFV Gc ectodomain crystallizes as a dimer, an assembly model has been proposed for the RVFV envelope in which Gc dimers are oriented horizontally respective to the viral membrane [43]. In contrast, the virion interior does not display a particular organization, in keeping with the absence of a matrix protein in all bunyaviruses. Thus, the Gn and Gc proteins are presented in a highly ordered fashion on the virion surface. In the following paragraphs it will be discussed how these proteins mediate viral entry into target cells and cause assembly and budding of progeny particles in infected cells.

### 2.2. Attachment Factors and Receptors

Phlebovirus entry into cells commences with binding of particles to components of the plasma membrane. For the purpose of this discussion, we will define attachment factors as such plasma membrane components, which interact with viral glycoproteins and modulate entry efficiency but are ultimately dispensable for infectious entry. In contrast, cellular factors that bind to viral glycoproteins and are essential for entry will be termed receptors. For hantaviruses, a role for β1-3 integrins in host cell entry has been reported and integrin choice was found to correlate with viral pathogenicity [44,45,46]. These observations suggest that protein-protein interactions may orchestrate cellular entry of hantaviruses, although direct binding of Gn and/or Gc to integrins remains to be demonstrated [31]. In contrast, glycan-protein interactions seem to play a prominent role in phlebovirus entry, as discussed below.

### 2.3. Dendritic Cell-Specific Intercellular Adhesion Molecule-3-Grabbing Non-Integrin (DC-SIGN) Facilitates Phlebovirus Entry into DCs

DC-SIGN is a calcium-dependent lectin expressed on DCs, certain tissue macrophages, megakaryocytes, a subset of B-cells, and platelets [47]. DC-SIGN recognizes mannose and fucose residues on cellular ligands and several pathogens [48], including human immunodeficiency virus (HIV) [49] and mycobacterium [50,51], and tetramerization of DC-SIGN is required for avid ligand binding. In the context of phleboviruses, it was shown that DC-SIGN facilitates entry of UUKV, RVFV, Punta Toro virus (PTV) and Toscana virus (TOSV) [52]. Subsequent work showed that DC-SIGN also facilitates entry of vectors pseudotyped with SFTSV and La Crosse virus (LACV) glycoproteins [53]. Phleboviruses are transmitted by arthropod bites, hence, skin and tissue DCs are amongst the first cells encountered by these viruses, suggesting that DC-SIGN could be important for viral transmission. Indeed, initial studies on dengue virus (DENV), another arbovirus, showed that DC-SIGN promotes DENV infection [54,55], although a subsequent report demonstrated that DENV infection of human skin cells is DC-SIGN-independent [56]. Interactions of phleboviruses with DC-SIGN depend on *N*-glycans located on Gn and/or Gc and DC-SIGN expression was shown to be required for DC infection by UUKV [52] and for SFTSV Gn/Gc-mediated transduction of these cells [53]. In addition, DC-SIGN expression was sufficient to render cell lines susceptible to phlebovirus entry [52,53]. Thus, DC-SIGN is a bona fide phlebovirus receptor, which, due to endocytosis signals in its cytoplasmic tail [57], promotes uptake of phleboviruses into cells [52]. In early endosomes, the virions dissociate from DC-SIGN and continue the degradative pathway to late endosomes [52], the location of membrane fusion. It is noteworthy that many cell lines susceptible to phlebovirus infection do not express DC-SIGN [52], indicating that these viruses most likely also use other receptors for infectious entry. Other lectins like the DC-SIGN-related protein DC-SIGNR (L-SIGN) and LSECtin were shown to promote entry of several viruses [58,59,60] and might also augment phlebovirus infection of certain cells. Indeed, a recent report demonstrated that DC-SIGNR, which shares 77% amino acid sequence identity with DC-SIGN but is expressed on different cells (endothelial cells of liver and lymph nodes), can markedly augment phlebovirus entry into cell lines which are otherwise barely susceptible [61]. In contrast to DC-SIGN, L-SIGN mainly promotes viral attachment but not uptake into cells [61] in keeping with the established concept that DC-SIGN but not DC-SIGNR functions as an endocytic receptor [62]. Thus, it is conceivable that DC-SIGNR promotes phlebovirus entry by concentrating virions onto the cell surface, thereby increasing interactions with a so far unidentified receptor. The presence of such receptor(s) is strongly suggested by the broad cell tropism of several phleboviruses and the relatively narrow cell and tissue expression of the lectins discussed above. Finally, it is noteworthy that DC-SIGN might promote SFTSV pathogenesis independent of its function as a viral receptor: SFTSV was shown to associate with platelets [63], which are known to express DC-SIGN and to capture HIV and potentially other viruses in a DC-SIGN-dependent fashion [64]. Moreover, SFTSV-platelet complexes were found to be taken up into macrophages [63], suggesting that DC-SIGN-dependent SFTSV interactions with platelets could contribute to removal of platelets from the circulation and thus to thrombocytopenia, a hallmark of SFTS.

### 2.4. Heparan Sulfate (HS) Proteoglycans Promote Phlebovirus Attachment

HS is a glycosaminoglycan (GAG), an unbranched polysaccharide composed of disaccharide repeats, which can be linked to a protein via *O*-glycosylation, resulting in the formation of a proteoglycan. Several viruses engage HS for entry into target cells. Analysis of cell lines with defined glycosylation defects revealed that HS, but not complex *N*-glycans, is required for efficient cellular entry of RVFV [65]. This observation was confirmed by enzymatic removal of HS and competition experiments with heparin. Moreover, evidence was obtained that *O*-sulfation of HS is essential for RVFV entry [65]. Viral interactions with HS are frequently charge-dependent and sequence analysis revealed clusters of basic amino acids on the P78 protein, which might interact with negatively charged sulfate groups on HS [65]. In contrast, potential HS binding sites on Gn or Gc were not identified. The P78 protein is one out of four translation products of the M genomic segment of RVFV and its translation efficiency seems to be cell line-dependent. While P78 is quite abundant in RVFV-infected insect cells, mammalian cells produce only small amounts of P78 [66]. As a consequence, purified RVFV virions derived from Vero E6 cells did not contain detectable amounts of P78 [66]. Moreover, P78 is dispensable for RVFV virulence in mice [67]. Therefore, the HS binding sites on RVFV produced in mammalian cells await further investigation. However, it is noteworthy that P78 protein is efficiently incorporated into RVFV produced in mosquito cells [66] and is required for viral dissemination in mosquitos [67]. Whether HS binding accounts for the important role of P78 in viral spread in mosquitos remains to be elucidated. A role of HS in RVFV entry was also identified within a screen of haploid cells for factors required for RVFV spread [68]. This study reported that HS-dependence for entry of RVFV did not result from cell culture adaptation since primary isolates were found to depend on HS for entry [68]. Moreover, the role of HS in RVFV entry was shown to be cell type-dependent and evidence was provided that Crimean Congo hemorrhagic fever virus (CCHFV) and Hantaan virus (HNTV) rely on HS for efficient entry while Andes virus (ANDV) does not [68]. Additionally, separate work showed that TOSV uses GAGs for efficient cell entry [69]. Whether GAGs serve as attachment factors or as receptors is unknown. However, the cell line dependence of the role of HS in RVFV entry in combination with the detection of residual infection in the absence of HS suggest that GAGs might serve as attachment factors rather than receptors.

### 2.5. Non-Muscle Myosin Heavy Chain IIA (NMMHC-IIA) Promotes SFTSV Entry

NMMHC-IIA is an actin binding motor protein that induces actin crosslinking and contraction and plays a role in cell migration, adhesion, and polarization [70]. Sun and colleagues showed that recombinant SFTSV-Gn bound to susceptible cell lines and identified NMMHC-IIA as a cellular binding partner of Gn [71]. Moreover, evidence was obtained that inhibition of NMMHC-IIA expression or blockade by antibodies reduces viral entry while directed expression can increase entry efficiency [71]. However, formal proof that directed expression of NMMHC-IIA renders otherwise entirely refractory cells susceptible to infectious SFTSV entry remains to be provided. Binding of SFTSV to cells augmented total expression of NMMHC-IIA and increased surface levels within minutes (the protein is normally localized in the cytoplasm), indicating that SFTSV manipulates NMMHC-IIA trafficking to ensure efficient entry [71]. Moreover, SFTSV might parasitize the documented role of NMMHC-IIA in endocytosis and phagocytosis to ensure its uptake into the cells [71]. Finally, it has been suggested that SFTSV interactions with NMMHC-IIA might directly contribute to viral pathogenesis [71]. Thus, point mutations in NMMHC-IIA were found to be associated with thrombocytopenia [72] and obstruction of normal NMMHC-IIA function by SFTSV might have similar effects. In addition, NMMHC-IIA, like DC-SIGN, might promote viral attachment to platelets followed by uptake and destruction of virus-platelet complexes by macrophages [71]. Collectively, NMMHC-IIA could play an important role in SFTSV entry, although evidence for a bona fide receptor function is still missing. It is noteworthy that other viruses also exploit NMMHC-IIA for cellular entry: NMMHC-IIA was identified as a receptor for herpes simplex virus type 1 (HSV-1) and HSV-1 attachment to cells was shown to increase surface levels of NMMHC-IIA [73].

### 2.6. Phlebovirus Uptake: Clathrin-Dependent and -Independent Mechanisms

A seminal study by Lozach and colleagues examined the steps ensuing receptor binding, uptake of virions into cells and membrane fusion. They could show that UUKV, upon attachment to plasma membrane indentations and filopodia, is taken up into the cell within minutes by a mechanism that is mainly independent of clathrin coats (clathrin-independent endocytosis, CIE) [74]. Internalized UUKV is then transported into early and late endosomes, where low pH triggers membrane fusion [74], as discussed below. Expression of vesicle-associated membrane protein 3 (VAMP3), which belongs to the vesicle synaptosome-associated protein receptor (v-SNARE) family of membrane proteins, was required for UUKV infection and virus particles were found in VAMP3-positive late endosomal compartments [75]. Thus, adequate intracellular transport of UUKV seems to depend on the documented role of VAMP3 in late vesicular trafficking events [76,77]. In addition, expression of histone deacetylase (HDAC) 8 was shown to be required for UUKV entry [78], likely because of its role in microtubule organization and endosomal maturation. These studies point towards an essential role of UUKV transport into late endosomes for infectious entry while the uptake mechanism requires further analysis. In this context, it should be noted that cell entry of RVFV depends on ribonuclease kappa (RNaseK) [79], which is also essential for the uptake of other pH-dependent viruses [79], and on caveolin-1-mediated endocytosis (CavME), while macropinocytosis and clathrin-mediated endocytosis (CME) do not play a role [80]. In contrast, vesicular stomatitis virus (VSV)-particles pseudotyped with SFTSV-Gn/Gc employ a clathrin-dependent mechanism for viral uptake, [53] and orthobunyaviruses also enter cells in a clathrin-dependent fashion [81,82]. Thus, different phleboviruses might use different uptake mechanisms for entry and potential strain and cell line-dependent differences remain to be investigated.

### 2.7. Virus-Cell Fusion and Its Inhibition

#### 2.7.1. Characteristics of Viral Membrane Fusion Proteins

Successful transport of virions into host cell endosomes and exposure to endosomal low pH initiates the last sequence of the phlebovirus entry cascade: the fusion of the viral envelope with an endosomal membrane. Three classes of viral proteins that can fuse viruses with cells have been identified. Class I membrane fusion proteins are usually oriented perpendicular to the viral membrane and α-helices are their predominant structural elements. In contrast, class II membrane fusion proteins frequently exhibit a parallel orientation relative to the viral membrane and a high content of β-sheets. Finally, class III membrane fusion proteins unite characteristics of both class I and II membrane fusion proteins [83,84]. All viral membrane fusion proteins have in common that a trigger, usually low pH or receptor binding (or a combination thereof), induces the membrane fusion reaction, which is facilitated by marked conformational changes in the glycoproteins. First, a fusion peptide or an internal fusion loop is propelled towards the target cell membrane and inserted into the bilayer. Then, a back-folding reaction brings the N- and C-termini of the glycoproteins and thus viral and cellular membranes into close contact and ultimately facilitates membrane fusion, allowing delivery of the viral nucleic acid into the host cell cytoplasm [83,84].

#### 2.7.2. RVFV Gc is a Class II Membrane Fusion Protein

A computational study examining bunyavirus glycoprotein sequences provided the first evidence that phlebovirus Gc proteins might be class II membrane fusion proteins. Thus, similarities were noted between the sequences of SFV Gc and the E1 protein of Sindbis virus (SINV) [85], a bona fide class II membrane fusion protein. Moreover, SFV Gc sequences potentially involved in membrane fusion were found to be conserved among bunyavirus Gc proteins [85]. Formal proof that phlebovirus Gc proteins are indeed class II membrane fusion proteins was provided by the elucidation of the structure of the ectodomain of RVFV Gc in the pre-fusion state. Gc was found to be organized into three domains with a fold characteristic of class II membrane fusion proteins [43]. An internal fusion loop was identified, a feature of all class II membrane fusion proteins, and the location of certain histidines in Gc suggested a role in pH sensing [43], as expected. Thus, protonation of histidines is known to trigger the membrane fusion reaction of many glycoproteins and histidine 1087 in RVFV Gc, which is required for infectivity [86], was located at the same site as histidines critical for triggering of other class II membrane fusion proteins by low pH [43]. Despite the apparent structural similarities between RVFV Gc and class II membrane fusion proteins, differences were noted. For instance, the interface between domains I and II in RVFV Gc is more extensive and potentially more rigid than that of other viral class II membrane fusion proteins. Moreover, RVFV Gc exhibits an increased number and altered localization of disulfide bridges as compared to other class II membrane fusion proteins [43]. These results suggest that phlebovirus Gc proteins might employ similar strategies as flavivirus E proteins and alphavirus E1 protein to facilitate membrane fusion, although subtle differences might exist.

#### 2.7.3. Low pH Triggers Membrane Fusion

The results discussed above suggest that the membrane fusion activity of phlebovirus Gc proteins is triggered by low pH upon transport of virions into endolysosomes. Indeed, treatment of target cells with lysosomotropic agents, which elevate intravesicular pH, blocks phlebovirus entry [53,86]. Moreover, exposure of Gn/Gc-expressing cells [87] or virions to low pH is sufficient to trigger Gc [74], and the ensuing conformational changes are irreversible, since triggering in the absence of target cells abrogates virus infectivity [86]. At present, no evidence has been reported that Gc proteins must first bind to a receptor or undergo proteolytic activation for subsequent triggering by low pH, although one report suggested that the activity of serine proteases in target cells is required for efficient SFTSV Gn/Gc-driven entry [53]. However, it is noteworthy that a trypsin-sensitive structure on target cell membranes might be required to support RVFV Gc-driven membrane fusion [87] and phospholipids with negatively charged headgroups were found to promote UUKV Gc-driven fusion in a liposome-based assay [88], indicating that specific components of the target cell membrane can impact fusion efficiency.

#### 2.7.4. Inhibition of Membrane Fusion by Interferon-Induced Transmembrane (IFITM) Proteins

The alteration of the biological properties of endolysosomal membranes is an innate defense against viral invasion. Thus, the IFITM 1–3 proteins are synthesized in response to viral invasion and block entry of several viral agents by modifying target cell membranes [89,90]. IFITM1 localizes at or close to the cell surface and blocks viruses from entering at these sites while IFITM2 and IFITM3 are found in endolysosomal compartments and inhibit viruses entering via these compartments [89]. In accordance with RVFV entry being dependent on endolysosomal low pH, expression of IFITM2 and IFITM3 was shown to block RVFV entry and more than half of the antiviral activity associated with IFNα treatment of target cells was found to be due to expression of these proteins [91]. How exactly IFITM proteins modulate membrane properties to inhibit viral entry is not clear, but alteration of membrane curvature and/or fluidity due to IFITM insertion and IFITM-IFITM interactions as possible mechanisms has been proposed [92,93].

## 3. Role of Gn and Gc in Phlebovirus Assembly

### 3.1. M Segment Coding Strategy and Expression of the Glycoproteins Gn and Gc

After fusion of viral and endosomal membranes the three viral genomic segments (L, M, and S) which are associated with the viral polymerase are released into the cytoplasm and primary transcription of negative-sense genomic RNA (gRNA) into mRNA is initiated [94]. Transcription and translation are tightly coupled, i.e., the translation of the viral proteins starts before the transcription of the mRNA is completed [95].

The two phlebovirus glycoproteins (like the glycoproteins of members of other *Bunyavirus* genera) are encoded on the M-segment in a single ORF [96,97,98,99]. They are synthesized as a precursor which is cotranslationally processed into the glycoproteins Gn and Gc [19,100,101,102,103]. The Gn/Gc precursor protein cannot be detected in phlebovirus-infected cells. Only after expression of M-segment-based plasmid constructs followed by pulse-chase immunoprecipitations, or after in vitro translation in the absence of microsomal membranes, does the precursor become visible [18,103,104]. In the presence of microsomal membranes, the precursor is rapidly cleaved, indicating cotranslational cleavage by a host factor during viral protein synthesis [103,104]. The host factor responsible for precursor cleavage is the signal peptidase complex located in the ER membrane [16,17].

Due to a signal sequence preceding Gn, the nascent precursor polypeptide chain is translocated from the cytoplasm into the ER. The Gn signal peptide is cleaved off by signal peptidase and the growing polypeptide chain is translocated into the ER lumen [19,105,106]. Two hydrophobic domains in the Gn/Gc precursor located in the C-terminal parts of Gn and Gc are inserted into the ER membrane and serve as transmembrane domains of Gn and Gc [97,106]. Additionally, Gn and Gc are separated by a third hydrophobic domain acting as internal signal peptide for Gc which is also cleaved by signal peptidase thus separating Gn from Gc [96,97,98,99]. 

Currently, the signal peptidase is the only host enzyme known to be required for the cleavage of the phlebovirus glycoprotein precursor [16,17]. This implies that the Gc signal peptide remains connected to the cytoplasmic C-terminal end of Gn, thereby acting as a second transmembrane domain for Gn. Indeed, for UUKV it has been shown that the Gc signal peptide is not removed from the cytoplasmic tail of Gn—at least not during glycoprotein synthesis and maturation [17]. However, it is not known if the Gc signal peptide is removed at another step of the viral life cycle.

**Figure 2:**
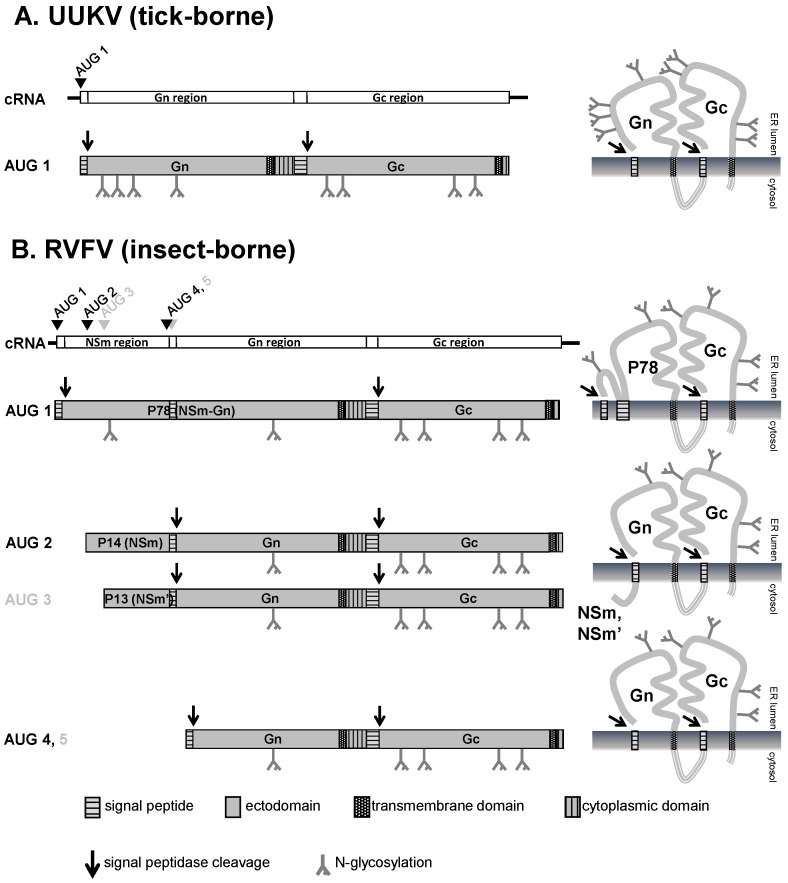
Coding and expression strategy of phlebovirus M-segments. Shown are the M-segments in antigenomic orientation (cRNA), the precursor glycoproteins and the membrane topology of the mature (glyco-) proteins. The antigenomic M-segment RNA serves as a template for viral transcription which results in a single mRNA. (**A**) UUKV as an example for tick-borne phleboviruses. The M-segment of tick-borne phleboviruses encodes only the two glycoproteins Gn and Gc. Translation of the mRNA yields one product, the Gn/Gc precursor. The precursor contains an N-terminal signal sequence preceding Gn and an internal signal sequence preceding Gc. Cleavage by the ER-associated signal peptidase complex yields Gn and Gc. Both Gn and Gc are glycosylated at *N*-glycosylation sites; (**B**) RVFV as an example for insect-borne phleboviruses. The M-segment of insect-borne phleboviruses encodes the non-structural protein NSm followed by the glycoproteins Gn and Gc. In case of RVFV translation initiation at different AUGs results in the expression of a nested set of polyproteins. Translation initiation at AUG 2 yields the NSm-Gn/Gc precursor protein. The precursor contains two internal signal sequences preceding Gn and Gc respectively. Cleavage by signal peptidase yields NSm, Gn and Gc. The Gn signal peptide acts as membrane anchor for NSm. Due to its membrane topology NSm is not glycosylated although it contains a potential *N*-glycosylation site. Translation at AUG 3 results in the expression of an N-terminal truncated NSm protein (NSm’) which is functionally equivalent to full-length NSm. Translation at AUG 1 yields the P78-Gc precursor protein. Signal peptidase cleaves the pre-protein after the signal sequences preceding NSm and Gc but not after the signal sequence preceding Gn which might act as membrane anchor instead. P78 is glycosylated at the *N*-glycosylation sites in the NSm and the Gn region. Note the different membrane topology of the NSm region in P78 (translation initiation at AUG 1) compared to NSm or NSm’ (translation at AUG 2 or AUG 3). Although P78 and Gc interact with each other, Gc might be unstable in the absence of Gn and therefore might be degraded in the ER. Translation at AUG 4 or 5 yields the Gn/Gc pre-protein. Signal peptidase cleaves the pre-protein after the signal sequences preceding Gn and Gc. Both Gn and Gc are *N*-glycosylated. The in vivo relevance of translation initiation at AUG 3 and 5 is not clear.

While the M-segment of tick-borne phleboviruses only encodes the glycoproteins Gn and Gc [36,53,105,107,108] the M-segment of insect-borne phleboviruses encodes an additional protein upstream of Gn termed NSm [97,102,104,105] (see Figure 2). Since all M-segment-encoded proteins are expressed from a single mRNA, an NSm-Gn/Gc precursor protein is produced in addition to the Gn/Gc precursor by differential use of an AUG triplet as start codon which is located upstream of the Gn start codons [99,109]. In the case of RVFV, another two AUG triplets—one upstream and one downstream of the NSm start codon—give rise to the expression of a nested set of polyproteins [101,104,110]. The polyprotein precursors are all cleaved by signal peptidase to generate the accessory proteins P78 (Nsm-Gn), P14 (NSm), and P13 (NSm’) in addition to the glycoproteins Gn and Gc [67,111]. The role of NSm proteins in the replication of insect-borne phleboviruses is not entirely clear. In vertebrate cells, the P14 protein of RVFV acts as an anti-apoptotic factor [112], however it is not required during viral replication in mammalian or mosquito cell cultures [113,114]. In vivo, P14 appears to be a virulence factor in mammals while P78 seems to be required for the dissemination in the mosquito vector [67], as discussed above. Mutational analysis revealed that the entire NSm region is dispensable for the proper synthesis and processing of the viral glycoproteins although both the NSm-Gn/Gc precursor and the Gn/Gc precursor can contribute to the synthesis of Gn and Gc [102,110,114].

Both Gn and Gc are type I transmembrane proteins, i.e., the N-terminus is orientated towards the ER lumen and the C-terminus is facing the cytoplasm (which corresponds to the interior of the virus after budding) and they span the lipid bilayer only once (although the signal peptide of Gc might serve as second transmembrane domain for Gn as described above) [17,105,115].

### 3.2. Post-translational Modifications and Subcellular Localization of Gn and Gc

Gn and Gc have a cysteine content of approximately 5% [98,105]. Positions of the cysteine residues are highly conserved among phleboviruses [99], indicating that extensive disulfide-bridge formation may occur and that the positions might be crucial for determining correct polypeptide folding. For Gn and Gc of UUKV it could be demonstrated that both proteins interact with protein disulfide isomerase (PDI) [116], an enzyme ubiquitously found in the ER which breaks up incorrectly formed disulfide bonds and catalyzes the formation of the correct ones leading to the mature, correctly folded three-dimensional protein structure. Other proteins involved in correct folding of UUKV Gn and Gc are the chaperones binding immunoglobulin protein (BiP), calnexin, and calreticulin [116,117] (Figure 1). 

Both Gn and Gc contain *N*-glycosylation sites (Asn–X–Ser or Asn–X–Thr) [18,98,109,118], but the exact number of these sites differs between the different phlebovirus species [97,99]. *N*-glycosylation occurs during protein synthesis in the lumen of the ER. Inhibition of *N*-glycosylation decreases the stability of Gn and Gc as demonstrated for the glycoproteins of PTV [119] and prevents the exit of the glycoproteins from the ER [100]. 

N-glycosylated and correctly folded Gn and Gc form non-covalently linked heterodimers in the ER [13]. The two glycoprotein molecules which associate as a heterodimer do not necessarily originate from the same precursor protein. In the case of UUKV, Gn matures significantly faster than Gc [116]. Therefore, newly synthesized Gn can only dimerize with Gc, which was synthesized earlier [116]. In contrast, in the case of PTV, heterodimers are formed by Gn and Gc molecules synthesized at the same time [119] suggesting that PTV and RVFV Gn and Gc maturate with similar kinetics [13,16].

An interesting feature of the glycoprotein heterodimers is their intracellular localization. After Gn/Gc dimerization the glycoproteins exit the ER and reach the Golgi apparatus [13,19,22,24,100,102,120]. In contrast to many other viral glycoproteins which are further transported to the plasma membrane the Gn/Gc heterodimers of phleboviruses (and all other bunyaviruses) are retained in the Golgi [13,20,105,120,121,122,123]. Consequently, bunyaviruses bud at the Golgi instead of the plasma membrane [24,124,125,126]. Mutational analysis revealed that only Gn, but not Gc, contains a Golgi retention signal [13,106,115,121,122,127]. The Golgi retention signal seems to be specific for each phlebovirus species since a conserved sequence for this signal could not be identified. In the case of UUKV, the sequence required for Golgi retention is entirely located in the cytoplasmic tail of Gn [115,121] while for RVFV and PTV the Golgi retention signal consists of the Gn transmembrane domain and the adjacent amino acids of the cytoplasmic tail [122,124,127]. As a consequence, all phlebovirus Gn proteins analyzed so far correctly localize to the Golgi in the absence of Gc [122,123,127]. However, Gc does not localize to the Golgi in the absence of Gn [106,122,123]. A lysine-based putative ER retention/retrieval signal is located in the short cytoplasmic tails of phlebovirus Gc proteins. In Gn/Gc heterodimers the ER retention signal of Gc is presumably masked by interaction with the cytoplasmic tail of Gn. Gc is therefore only targeted to the Golgi as long it is associated with Gn. An amino acid alignment of the extreme C-termini of Gc from viruses belonging to the genera *Phlebovirus*, *Hantavirus*, and *Orthobunyavirus* revealed that the lysine at position −3 is conserved across these genera [126]. Furthermore, in some phlebovirus Gc proteins, the conserved lysine is part of a KKXX motif which is the classical ER retention motif for transmembrane proteins [128,129]. Indeed, most phlebovirus Gc proteins are retained in the ER when expressed alone, although PTV Gc has been shown to reach the plasma membrane despite the presence of the conserved lysine at position −3 [106]. A possible explanation might be the fact that ER retrieval signals are not always functional when they are located in short cytoplasmic tails or near amphipathic helices. For simian immunodeficiency virus (SIV) envelope glycoprotein (Env) mutants harboring an additional KKXX-motif in the cytoplasmic tail, it has been demonstrated that only mutants with a cytoplasmic tail longer than 13 amino acids were retained in the ER. In contrast, SIV Env mutants with a cytoplasmic tail length of 13 amino acids or less were transported to the cell surface [130]. 

In Gn/Gc heterodimers, the conserved lysine in Gc additionally seems to contribute to Golgi retention since heterodimers of UUKV wild-type Gn and Gc with mutations at position −3 were retained in the ER [126]. Furthermore, growth of recombinant RVFV was severely impaired when the conserved lysine in the Gc tail was mutated, because the mutation led to a mislocalization of Gn at the cell surface [124]. Interestingly, the glycosylation pattern of Gn and Gc incorporated into virions reflects their localization signals. Gn carries mostly *N*-linked oligosaccharides of the complex type, indicating extensive oligosaccharide processing in the Golgi, while Gc glycosylation is mainly of the high-mannose or hybrid type [18,118,131,132], in keeping with predominant localization of Gc in the ER. In the case of UUKV, however, the differences in Gn and Gc glycosylation might not result from differential transit of Gn and Gc through the host cell. Instead, steric occlusion seems to prevent processing of *N*-glycans attached to UUKV Gc [133].

### 3.3. The Role of the Cytoplasmic Tails of Gn and Gc in Virus Assembly and Budding

As mentioned above, the cytoplasmic tail of phlebovirus Gc proteins is very short (e.g., only five amino acids for UUKV) while the cytoplasmic tail of Gn is much longer (e.g., 81 amino acids for UUKV) [105]. The extended length is associated with additional biological functions: the Gn cytoplasmic tail not only contains the Golgi localization signal but is also involved in the initiation of the budding process and the packaging of ribonucleoproteins (RNPs) into virus particles [134]. For UUKV it could be demonstrated that mutation of a di-leucine motif in the cytoplasmic tail of Gn abolished the budding of virus-like particles, although the UUKV glycoproteins were correctly localized to the Golgi [126]. However, the motif required for budding seems to be specific for UUKV since not all phlebovirus Gn proteins contain the di-leucine motif. In the case of RVFV, the di-leucine motif is replaced by phenylalanine and isoleucine [124]. Although mutations of these amino acids affected the release of RVFV virus-like particles, the growth of recombinant virus carrying the mutations was only slightly diminished [124]. Furthermore, for UUKV the Gn and Gc glycoproteins are sufficient for efficient formation and release of virus-like particles [28] whereas for RVFV the formation of virus-like particles in the absence of RNP is inefficient [27].

A distinct feature of phleboviruses (and all other bunyaviruses) is the lack of a matrix protein that typically acts as an anchor between the virus envelope and the genetic core, the RNP [135]. Instead, the cytoplasmic tail of phlebovirus Gn proteins is endowed with matrix protein-like functions. For UUKV, the most C-terminal residues of the cytoplasmic tail of Gn are essential for the incorporation of RNP into virus-like particles [134]. In contrast, for RVFV the N-terminal part of the cytoplasmic tail of Gn is essential [27,124]. In the case of RVFV, the cytoplasmic tail of Gn can bind and package the viral polymerase and the nucleoprotein independently, but the efficient release of virus-like particles requires the nucleoprotein-encapsidated genome-like RNA [27]. For UUKV and PTV it has been observed that interaction of nucleoprotein and glycoproteins only occur in the Golgi and not in the ER although in both compartments the cytoplasmic tail of Gn should be accessible for the nucleoprotein which is synthesized in the soluble fraction of the cytoplasm [22,24,100,120]. Obviously, local accumulation of glycoproteins in the Golgi is a prerequisite for efficient binding of the nucleoprotein or the RNP. It can therefore be assumed that the interaction of RNP and Env proteins is the driving force for the morphogenesis and the budding of phlebovirus particles in the Golgi. When the encapsidation of the ribnucleoproteins and budding of newly formed virus particles in the Golgi are completed, virion containing vesicles are transported via the exocytic pathway to the plasma membrane where the virus particles are released [136].

## 4. Conclusions

Considerable progress has been made over the last three decades in understanding the role of the glycoproteins in phlebovirus entry. In particular, the finding that phlebovirus Gc proteins are class II viral membrane fusion proteins provided important insights into the membrane fusion reaction and imaging approaches allowed to elucidate the cell biology of phlebovirus entry. Moreover, several attachment factors were identified that might explain viral tropism. However, the expected key determinant of entry and cell tropism, the receptors used by phleboviruses, remain largely elusive. In addition, potential differences between host cell entry of tick-borne and insect-borne phleboviruses and differences in entry into vectors and host cells await further investigation. The emergence of new pathogenic tick-borne phleboviruses, namely Heartland virus (HRTV) and SFTSV, highlights the importance for this type of research. Although the processing of phlebovirus glycoproteins by signal peptidase is a pivotal step of glycoprotein maturation, only limited experimental data concerning this process is currently available. The subsequent steps in phlebovirus glycoprotein maturation, i.e., disulfide bond formation and *N*-glycosylation are even less well characterized. Furthermore, the mechanism of how glycoproteins and RNPs interact during virus assembly is poorly understood. New insights into these topics, together with a better understanding of the phlebovirus entry process, might provide the basis for the rational design of effective countermeasures against highly pathogenic phleboviruses.

## Abbreviations

The following abbreviations are used in this manuscript:

ANDV	Andes virus
BiP	Bindung immunoglobulin protein
CavME	Caveolin-1-mediated endocytosis
CIE	Clathrin-independent endocytosis
CME	Clathrin-mediated endocytosis
CNX	Calnexin
CCHFV	Crimean Congo hemorrhagic fever virus
DC	Dendritic cell
DC-SIGN	Dendritic cell-specific intercellular adhesion molecule-3-grabbing non-integrin
Env	Envelope glycoprotein
ER	Endoplasmic reticulum
GAG	Glycosaminoglycan
gRNA	genomic RNA
HDAC 8	Histone deacetylase 8
HRTV	Heartland virus
HIV	Human immunodeficiency virus
HNTV	Hantaan virus
HS	Heparan sulfate
IFITM	Interferon-induced transmembrane protein
L-SIGN	Liver/lymph node-specific intercellular adhesion molecules-3 grabbing non-integrin
LACV	La Crosse virus
MФ	Macrophage
NMMHC-IIA	Non-muscle myosin heavy chain IIA
ORF	Open reading frame
PDI	Protein disulfide isomerase
PTV	Punta Toro virus
RNaseK	Ribonuclease kappa
RNP	Ribonucleoprotein
RVFV	Rift Valley fever virus
SFV	Sandfly fever virus
SFTSV	Severe fever with thrombocytopenia virus
SIV	Simian immunodeficiency virus
TOSV	Toscana virus
UUKV	Uukuniemi virus
v-SNARE	Vesicle-soluble NSF attachment protein receptor
VAMP3	Vesicle-associated membrane protein 3
VSV	Vesicular stomatitis virus
